# Paul of Aegina (ca 625-690 AD): Operating on All, from Lymph Nodes in the Head and Neck to Visceral Organs in the Abdomen

**DOI:** 10.7759/cureus.7287

**Published:** 2020-03-16

**Authors:** Dimitrios Papapostolou, Anastasios Karandreas, Evagenlos Mavrommatis, Konstantinos Laios, Theodore Troupis

**Affiliations:** 1 Anatomy, School of Medicine, National and Kapodistrian University of Athens, Athens, GRC; 2 Surgery, School of Medicine, National and Kapodistrian University of Athens, Athens, GRC

**Keywords:** alexandria, surgical anatomy, tonsillectomy, cauterization, cicatrisation

## Abstract

In the seventh century AD, a native of the island of Aegina, a brilliant surgeon, Paulus Aegineta, practiced surgery in Byzantium. Able to perform a wide variety of surgical operations, he summarized his experience and gathered the available knowledge to compose his masterpiece "Medical Compendium." He was credited as the first to operate on the tonsils and the lymphatic system of the lower cervical region and as one of the pioneers to cut the abdominal wall. Fond of the cauterization method, an expert in disinfection and pain palliation, he had presented supreme skills, becoming one of the most important figures in the history of medicine.

## Introduction and background

The human body in the ancient Hellenic world was considered the vessel of the soul and was depicted mainly in marble as a perfect figure, an apotheosis of the human figure, the measure of all, including beauty and divine. In addition, the body of the dead was also sacred for the ancient Greeks and should not be touched due to religious and ethical considerations [1]. Anatomy was practiced with the help of animals or, sometimes, on terracotta figurines. Surgery was born since the early days of human evolution, mainly through the field of hunting or on the battlefield. The first written example of battle surgeons is that of Homer's Iliad, where Machaon, the son of Asclepius, a brave warrior-surgeon became a god and was worshiped for his healing skills. Surgery was systematized by Hippocrates while a series of minor surgical interventions was practiced inside Asclepieia. Various operations were performed in daily practice, mainly superficial, as body cavities presented a fatal danger for the patient [2-3]. Cauterization provided hemostasis and small tumor and tissue destruction. Disinfectants were also in use, while the surgeon had a medical box with his drugs and instruments in place. Cranial trepanation, hepatic diseases, breast cancer, battle wounds, and orthopedics were in the surgeons' field [2,4].

Surgery in the Hellenic peninsula reached a point of success, adequate considering the era, while later in Byzantine times, it did not evolve as excepted. However, some majestic scholars became the exception. Among them, Paul of Aegina (seventh century AD), who practiced surgery in its fullness, operating all that he could and all that he couldn't. This historical vignette tries to present Paul's figure and some of his surgical techniques in the head and neck and abdominal areas.

## Review

Paul of Aegina: life and work

Paul of Aegina or Latinized Paulus Aegineta (Greek: Παῦλος Αἰγινήτης) was a native of a small island, Aegina, one of the Saronic islands near the city-state of Athens, only 16 nautical miles away from its port Piraeus. He is considered to be the last of the Greek compilers in Alexandrian School, a Greek Medical school in Egypt, which was very famous in anatomy learning through human dissections. Little is known for his life but it is almost certain that he had exercised medicine in Alexandria, just before its destruction by the Arabs during the seventh century AD. He was an expert surgeon, a brilliant scholar, and a prolific writer. He had composed an encyclopedic treatise titled "Medical Compendium" (Greek: Πραγματεία Ιατρικής) in seven books. As his work was unrivaled in its accuracy and completeness, Arabs translated it and adopted it in their works. In this way, Paul strongly influenced almost all physicians in the Mediterranean and Arabian world [5]. He was known by the Arabs as the "Obstetrician" and by the Byzantines as the "Peregrinator" (Greek: περιοδευτής), and "Iatrosophistis" (Greek: ιατροσοφιστής, meaning an authority in medicine). His skills in practicing a plethora of innovative surgical operations testify that as all medico-philosophers of Greek antiquity, he had traveled around the known world. He is believed to have written two monographs titled "On the therapy and treatment of the child" and "On Gynaecology" [5-6]. His work was mostly affected by the Galenic views, as Galen (ca 129-210 AD) was considered an authority in Western medicine [7]. Great medical minds, such as Persians polymath Rhazes (854-925 AD), physician Haly Abbas (c. 10th century AD), medical philosopher Avicenna (980-1037 AD), the majestic Arab physician Albucasis (936-1013 AD), and Italian anatomist and surgeon Fabricius ab Aquapendente (1537-1619 AD), had been all Paul's admirers. His work enjoyed future publications as those in Venice during 1528 and in London between 1844 and 1847 [8].

Surgery, skills, and innovations

Surgical knives, scalpels, various dioptras, psalis, hedrodiastoleus, mochliskos, ostagra, cauterion, motos moloubus, catheters, metrechytes, agkystra, tricholabis, embryoulkos, staphylagra, osteotomes, spathomele, and cyathiscomele are some of the surgical instruments that were available to Paul for performing his operations [2,9]. Antisepsis as a protocol was known since the era of Hippocrates (ca 460-370 BC) and boiled rainwater, salt, hot seawater, copper, tar, resin, myrrh, aloe, and various perfumes and minerals had been being placed to clear cutaneous wounds or skin itself [10-14]. As for pain during the operation, Paul used mandragoras (Greek: μανδραγόρας; it contains scopolamine) or its combinations with Morus alba (Greek: οπός μούρων) and hedera (Greek: οπός κισσού), known sedatives since Classical Greece. Furthermore, he had described the effect produced by mandrake, "when someone drinks mandragor, stupor appears, with loss of strength, and a strong inclination to sleep, called lethargy" [14]. His vivid descriptions of a series of surgical techniques like tracheotomy, tonsillectomy (amygdalectomy), catheterization of the bladder, transurethral lithotomy, inguinal herniotomy, cauterization, abdominal paracentesis for ascites, finger amputation, cancerous mastectomy, uterus cancer, and many other surgical procedures, including reduction of breast size (aesthetic surgery) and cataract lead us to the conclusion that his skills were unparallel [14-16].

Operating from head to toe, Paul was aware of tonsillectomy and direct laryngoscopy, which had been firstly performed by Hippocrates, and later upgraded by Celsus (ca 25 BC-50 AD) who used his fingers to remove the tonsils and by Aetius of Amida (ca mid-fifth to sixth century AD) who introduced a method using a hook and knife [14,17]. Paul had altered the procedure by scraping the mucous membrane with one of his fingers to enucleate the tonsil, while with his other hand, holding a forceps (Greek: αγκυλοτόμος), was able to completely extirpate it [14,17-18]. The lymphatic system was described by the distinguished members of the Alexandrian School Herophilos (ca 335-280 BC) and Erasistratus (ca 304-250 BC), although controversy exists regarding whether or not the anatomical structures being mentioned were actually lymph vessels. Nevertheless, Paul has been credited as the first to discover, describe, and operate on infected lymph nodes in the lower cervical region. It seems that during his tonsillectomy procedures, he had encountered and detailed local lymph nodes [19-20].

The abdominal cavity was presented as the most difficult area to be operated. Paul made commentaries on penetrating wounds and arrows and other weapons from extraction from fleshy parts such as the abdomen. For abdominal infections, such as those of the spleen and liver, he recommended surgical cauterization and draining of the ascites [8]. He is considered among the pioneers to confront abdominal distention [21]. Paul was able to distinguish three types of hernias: enteroceles, which are scrotal hernias, epiploceles, when the scrotum contained only the omentum, and bubonocele when hernia did not descend into the scrotum. During his herniotomy operations, he recommended the removal of the testicles and cauterization of the tissues to boost cicatrization of the area and stop local bleeding [22].

## Conclusions

Paul of Aegina mastered surgery in Alexandria, Egypt, a country with an inclination toward and tradition of surgical anatomy. His supreme ability to operate on all areas of the human body made him the founder of a new era of surgery. He had used a variety of innovative techniques, influencing physicians of both the Christian and Arabic world.
